# Investigation of magnetically controlled water intake behavior of Iron Oxide Impregnated Superparamagnetic Casein Nanoparticles (IOICNPs)

**DOI:** 10.1186/s12951-014-0038-4

**Published:** 2014-10-04

**Authors:** Anamika Singh, Jaya Bajpai, Anil Kumar Bajpai

**Affiliations:** BMRL, Department of Chemistry, Government Model Science College, Jabalpur, India

**Keywords:** Casein, IOICNPs, Swelling behaviour, pH sensitive, Magnetic drug targeting

## Abstract

Iron oxide impregnated casein nanoparticles (IOICNPs) were prepared by in-situ precipitation of iron oxide within the casein matrix. The resulting iron oxide impregnated casein nanoparticles (IOICNPs) were characterized by Scanning electron microscopy (SEM), Transmission electron microscopy (TEM), X-ray photoelectron spectroscopy (XPS), Fourier transform infrared (FTIR), Vibrating sample magnetometer (VSM) and Raman spectroscopy. The FTIR analysis confirmed the impregnation of iron oxide into the casein matrix whereas XPS analysis indicated for complete oxidation of iron (II) to iron(III) as evident from the presence of the observed representative peaks of iron oxide. The nanoparticles were allowed to swell in phosphate buffer saline (PBS) and the influence of factors such as chemical composition of nanoparticles, pH and temperature of the swelling bath, and applied magnetic field was investigated on the water intake capacity of the nanoparticles. The prepared nanoparticles showed potential to function as a nanocarrier for possible applications in magnetically targeted delivery of anticancer drugs.

## Introduction

The concept of magnetic drug targeting (MDT) simply entails retaining specially designed magnetic drug nanocarriers at a specific site in the body using an externally applied magnetic field. The two key properties for an effective nanocarriers are (a) efficient targeting to specific tissue and cells, and (b) avoiding rapid clearance (i.e. remaining in circulation) for a significant amount of time to increase particle uptake in target tissue. Circulation time, targeting, and the ability to overcome biological barriers depend on the shape (e. g, aspect ratio), chemical coating and size of the nanoparticles [1]. Thus a precise control over the shape and size of nanoparticles is a challenging task and must be addressed in order to achieve high performance drug delivery systems.

In order to achieve an efficient MDT, various polymeric/inorganic hybrid materials have been suggested that offer unique properties because of their small size, limited toxicity [2], low production cost, ease of separation and detection [3]. The magnetic nanoparticles often tend to form large aggregates owing to the strong magnetic dipole–dipole attractions among particles. To improve their chemical stability and biocompatibility, the surface of magnetic nanoparticles have been modified with various surfactants [4] or biopolymer compounds having multiple functional groups capable of binding to the particle surfaces (multidentate ligands). The rational for using magnetic nanoparticles to tumor targeting is based on the fact that nanoparticles will be able to deliver a concentrate dose of drug in the vicinity of the tumor targets via the enhanced permeability and retention effect or active targeting by ligands on the surface of nanoparticles [5]. Moreover, the extent of drug loading onto the nanoparticles greatly depends on the hydrophilic nature of the biopolymer also and, therefore, proteins could be an excellent option to design such magnetic nanocarriers.

Use of milk proteins, like caseins, in drug delivery applications is relatively a new trend and this is mainly due to its amphiphilic nature that allows them to interact with both the drug and solvent. In fact, caseins may be regarded as block copolymers with high level of hydrophilic and hydrophobic amino acid residues and thus they exhibit a strong tendency to self assemble into spherical micelles. Moreover, thier biodegradability, not-toxicity, metabolizablity and feasibility to surface modification enable them to interact with the targeting ligand. In an study, the complexation of curcumin with β-casein micelles increased the solubility of curcumin at least 2500-fold with enhanced curcumin cytotoxicity to a human leukemia cell line [6]. Shapira et al. [7] showed that β-CAS micelles could entrap and deliver hydrophobic chemotherapeutics such as mitoxantrone and paclitaxel, allowing them to be thermodynamically stable in aqueous solutions for oral delivery applications. Thus, the hydrophobic and hydrophilic domains of casein are responsible not only for their water sorption capacity, but also for the nature and type of drug to be encapsulted in the casein nanoparticles.

Thus, motivated by the pharmaceutical specialities of the casein protein the authors were pused to undertake a systemic investigation of synthesis and characteriztion of magnetic casein nanoparticles and their water sorption behavior to judge their suitability in designing swelling controlled and magnetic mediated drug delivery system. The present study aims at designing controllable size iron oxide impregnated casein nanoparticles (IOICNPs) by co-precipitation of iron salts within the casein nanoparticles matrix. As the It is also proposed to characterized the so prepared IOICNPs by various analytical techniques and investigate their water sorption potential in the presence of applied magnetic field.

## Experimental

### Materials

Casein was purchased from Merck, Mumbai, India and used without any pretreatment. FeCl_2_. H_2_O, FeCl_3_.6H_2_O, glutaraldehyde (used as a crosslinker) were obtained from Loba Chemie, Mumbai, India. Toluene was obtained from Sigma Aldrich Co., USA, and used for preparing oil phase. Other chemicals like acetone, NaOH etc. were of analytical reagent (AR) grade and double distilled water was used throughout the experiments.

### Preparation of Iron oxide impregnated casein nanoparticles (IOICNPs)

Preparation of magnetic casein nanoparticles consists of a two steps process. In the first step the casein nanoparticles are prepared by emulsion crosslinking method while in the second one iron oxide nanoparticles are impregnated within casein nanoparticles matrix by in situ precipitation.

### Preparation of casein nanoparticles (CNPs)

In order to prepare CNPs the microemulsion crosslinking method was adopted as described in literature [8]. In brief, an aqueous phase was prepared by dissolving known amount of casein in 1% NaOH whereas toluene was used to prepare the oil phase. The above two solutions were mixed with vigorous shaking (shaking speed 1000 RPM, 5 L capacity, Remi, India) for 30 min and to this emulsion 1 mL glutaraldehyde was added as crosslinker with constant stirring. The crosslinking reaction was allowed to take place for 30 min at room temperature (30°C) and H_2_SO_4_ was added to the solution for the solidification of particles. The nanoparticles were cleaned by washing them thrice with acetone and stored in air-tight polyethylene bags.

### Impregnation of Iron oxide in to the casein nanoparticles

The dried CNPs were placed in an aqueous mixture of Fe^2+^ and Fe^3+^ chloride salts at 1:2 molar ratio and allowed to swell for 24 h so that both Fe^2+^ and Fe^3+^ ions were entrapped in to the biopolymer matrix. Prior to putting them in salt solution, a dry stream of N_2_ was flushed for at least 15 min to control the reaction kinetics, which is strongly related to the oxidation speed of iron species. Bubbling nitrogen gas through the solution not only protects critical oxidation of the magnetite but also reduces the particle size [9]. The ferrous and ferric ions loaded casein nanoparticles were added to NaOH solution for a definite time period so that magnetite is precipitated within the biopolymer matrix according to the following chemical reactions [10].

1 $$ \begin{array}{l}\mathrm{F}{{\mathrm{e}}^2}^{+}+2\mathrm{O}\mathrm{H}\hbox{-} \to \mathrm{F}\mathrm{e}\ {\left(\mathrm{O}\mathrm{H}\right)}_2\\ {}\mathrm{F}{{\mathrm{e}}^3}^{+}+3\mathrm{O}\mathrm{H}\hbox{-} \to \mathrm{F}\mathrm{e}\ {\left(\mathrm{O}\mathrm{H}\right)}_3\\ {}\mathrm{F}\mathrm{e}{\left(\mathrm{O}\mathrm{H}\right)}_2+2\mathrm{F}\mathrm{e}{\left(\mathrm{O}\mathrm{H}\right)}_{3+}\mathrm{NaOH}\to \mathrm{F}{\mathrm{e}}_3{\mathrm{O}}_4+4{\mathrm{H}}_2\mathrm{O}\end{array} $$

Furthermore, for synthesis of nanoparticles with either the ferrous or ferric ion alone, the chemical reactions pass through different mechanisms as shown in Equations (2) and (3), respectively.

2 $$ \begin{array}{l}\mathrm{F}{{\mathrm{e}}^2}^{+}+2\mathrm{O}{\mathrm{H}}^{\hbox{-}}\to \mathrm{F}\mathrm{e}\ {\left(\mathrm{O}\mathrm{H}\right)}_2\\ {}3\mathrm{F}\mathrm{e}\ {\left(\mathrm{O}\mathrm{H}\right)}_2+0.5{\mathrm{O}}_2\to \mathrm{F}\mathrm{e}\ {\left(\mathrm{O}\mathrm{H}\right)}_2+2\mathrm{FeOOH}+{\mathrm{H}}_2\mathrm{O}\\ {}\mathrm{F}\mathrm{e}{\left(\mathrm{O}\mathrm{H}\right)}_2+2\mathrm{FeOOH}\to \mathrm{F}{\mathrm{e}}_3{\mathrm{O}}_4+2{\mathrm{H}}_2\mathrm{O}\end{array} $$

3 $$ \begin{array}{l}\mathrm{F}{{\mathrm{e}}^3}^{+}+3\mathrm{O}{\mathrm{H}}^{\hbox{-}}\to \mathrm{F}\mathrm{e}\ {\left(\mathrm{O}\mathrm{H}\right)}_3\\ {}\mathrm{F}\mathrm{e}\ {\left(\mathrm{O}\mathrm{H}\right)}_3\to \mathrm{Fe}\mathrm{OOH}+{\mathrm{H}}_2\mathrm{O}\\ {}12\mathrm{FeOOH}\to 4\mathrm{F}{\mathrm{e}}_3{\mathrm{O}}_4+6{\mathrm{H}}_2\mathrm{O}\end{array} $$

These two mechanisms provide a larger particle size as compared to the case of the mixture of ferrous and ferric ions because the particle size of magnetite also depends on the nature of the intermediate form. Since the methods adopted require longer reaction times for the transformations of ferric ions and ferrous ions so that the intermediates can continuously grow. However, care must be taken while adding NaOH because according to the thermodynamics of this reaction, a complete precipitation of Fe_3_O_4_ occurs in the range of 9 to 14 pH and in molar ratio of 1:2 for Fe^2+^: Fe^3+^ under a non oxidizing oxygen free environment. Otherwise, Fe_3_O_4_ might get oxidized as,

4 $$ \mathrm{F}{\mathrm{e}}_3{\mathrm{O}}_4 + 0.25{\mathrm{O}}_2 + 4.5{\mathrm{H}}_2\mathrm{O}\to 3\mathrm{F}\mathrm{e}\ {\left(\mathrm{O}\mathrm{H}\right)}_3 $$

**Figure 1:**
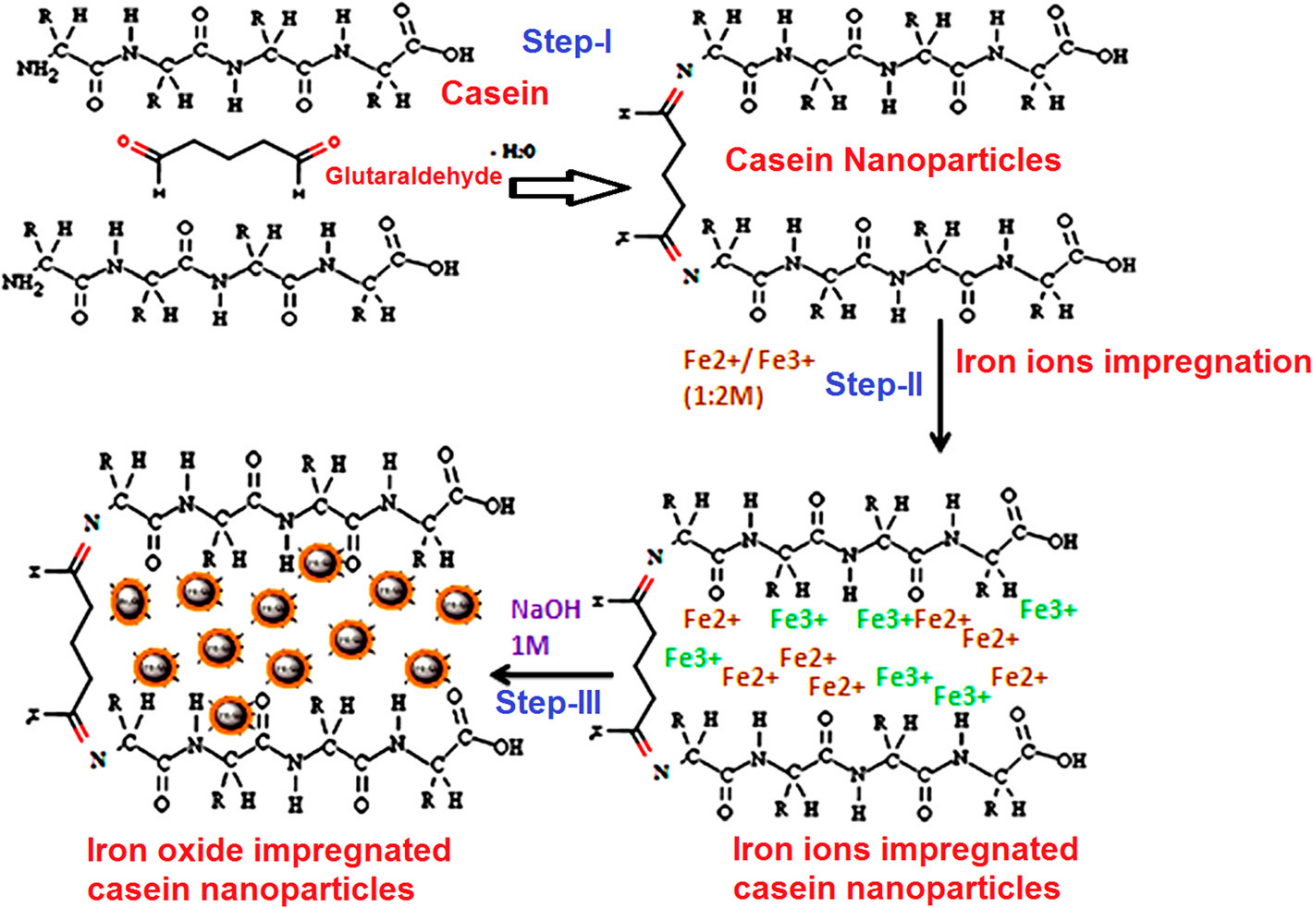
**Schematic formation of IOICNPs (Step-I) Formation of casein nanoparticles by crosslinking of casein macromolecules by reaction with glutaraldehyde.** (Step-II) Impregnation of iron ions within the casein nanoparticls network by swelling in iron salts solution. (Step-III) In situ precipitation of iron oxide within the casein nanoparticles matrix to yield IOICNPs.

The change in color of the casein nanoparticles from orange to dark brown also confirms the formation of oxides of iron. The prepared nanoparticles were washed, dried at room temperature and stored in airtight polyethylene bags. The chemical reaction is shown in Figure 1. The percentage impregnation of iron oxide was calculated using following equation.

5 $$ \mathbf{Impregnation}\left(\%\right)=\frac{\mathbf{Wimpregnated}\mathbf{\hbox{-}}\mathbf{Wdry}}{\mathbf{Wdry}}\times \mathbf{100} $$

In fact, the process of formation of iron oxide involves the diffusion of ferrous/ferric ions into the polymer matrix and their subsequent in situ precipitation in an alkaline medium. It is therefore expected that the higher is the water uptake greater will be, the iron oxide formation [11].

## Characterization

### FTIR Spectral analysis

The FTIR spectra of casein and IOICNPs were recorded on a FTIR- 8400, Shimadzu Spectrophotometer. Samples for the spectral analysis were prepared by mixing nanoparticles and KBr in 1:10 proportion and the spectra were obtained in the range of 4000 to 400 cm^−1^ with a resolution of 2 cm^−1^.

### SEM analysis

Morphological studies of cross-linked CNPs and IOICNPs were performed using SEM, Philips 515, fine coater (Philips, Eindhoven, The Netherlands). Drops of the polymeric nanoparticles suspension were placed on a graphite surface and freeze-dried. The sample was then coated with gold by ion sputter at 20 mA for 4 minutes, and observations were made at 10 kV.

### TEM analysis

The size and morphology of the nanoparticles were determined by conducting TEM analysis of casein and IOICNPs on Morgagni 268-D Transmission Electron Microscope with an accelerating voltage of 80.0 kV. The samples for TEM measurements were prepared by dispersing a drop of the sample solution on Formvar-coated C grids.

### Raman spectral analysis

In order to investigate the impregnation of iron oxide nanoparticles in to the matrix of casein nanoparticles, Raman spectroscopy was used and the spectra were obtained in the range of 200–1800 cm^−1^. The characteristic peak position of magnetite (Fe_3_O_4_) and its possible oxidation product maghemite (У-Fe_2_O_3_) and hematite (α- Fe_2_O_3_) were determined in the Raman region of 100–1200 cm^−1^. For correct assignment of the band positions and phase identification present in the samples, combined Raman data were used for key iron oxides bands. The Raman spectra of casein and IOICNPs were recorded on a Micro Raman Spectrometer, Jobin Yvon Horibra LABRAM-HR.

### VSM analysis

The magnetization versus magnetic field measurements (M–H first magnetization curve and hysteresis loop) at 300 K, for the IOICNPs (powder sample) were done on 14 T PPMS- vibrating sample magnetometer.

### XPS analysis

The samples were also analyzed by X-ray photoelectron spectroscopy (XPS) on a modified laser ablation system, Riber LDM-32, using a Cameca Mac3 analyzer. Photoelectron spectra were collected by acquiring data for every 1.0 eV with an energy resolution of 3 eV. Narrow-scan photoelectron spectra were recorded for C 1 s, N 1 s, O 1 s, and Fe 2p by acquiring data for every 0.2 eV and the energy resolution was 0.8 eV.

### In vitro cytotoxicity test

In order to determine in vitro cytotoxicity of the prepared materials test on extract method (ISO10993-5,2009) was applied. In brief, a test sample of the nanoparticles, negative control and positive control in triplicate were placed with subconfluent monolayer of L-929 mouse fibroblast cells. After incubation of cells with test samples at 37 ± 1°C, for 24 h, cell culture was examined microscopically for cellular response around and under the test samples.

### Water sorption capacity

The extent of swelling of CNPs and IOICNPs in both presence and absence of magnetic field were determined by a conventional gravimetric procedure as reported in the literature [12]. The swelling ratio was determined by the following equation:

6 $$ \mathbf{Swelling}\ \mathbf{Ratio}=\frac{\mathbf{Weight}\;\mathbf{of}\;\mathbf{s}\mathbf{wollen}\;\mathbf{nanoparticles}\left(\mathbf{W}\mathbf{s}\right)}{\mathbf{Weight}\;\mathbf{of}\;\mathbf{d}\mathbf{rynanoparticles}\left(\mathbf{W}\mathbf{d}\right)} $$

The amount of water imbibed by the sample provides information about the hydrophilic nature of the material, which is one of the criterions for biocompatibility.

### Effect of pH

The effect of pH on swelling of the nanoparticles was studied by preparing solutions over the pH range 1.8 to 9.0, and the desired pH was adjusted with the help of 0.1 M HCl and 0.1 M NaOH solutions. The pH was determined on a digital pH meter (Systronics, No. 362, Ahmadabad, India).

### Effect of temperature

The effect of temperature on swelling of the nanoparticles was studied by varying temperature of the swelling medium in the range of 10° to 40°C.

### Swelling studies in physiological fluid

In order to study the swelling of nanoparticles in simulated biological media, the following aqueous fluids (100 mL) were prepared: Saline water (0.9 g NaCI), synthetic urine (0.8 g NaCl, 0.10 g MgSO_4_, 2.0 g urea, 0.6 g CaCl_2_), urea 5.0 g, and D-glucose 5.0 g.

### Statistical analysis

All experiments were done at least thrice and Figures and data have been expressed along with their respective error bars and standard deviations, respectively.

## Results and discussion

### Effect of composition on impregnation

Impregnation of iron oxide into the polymer matrix is a result of inclusion of ferrous/ ferric ions into the polymer matrix and their subsequent *in situ* precipitation in alkaline medium. The impregnation process basically depends on the swelling capacity of the biopolymeric network which, in turn, varies as a function of chemical composition of the CNPs. Among various structural factors influencing water sorption capacity of a CNPs, the ratio of hydrophilicity to hydrophobicity plays a key role in determining swelling characteristic of the matrix. In the present study, the prepared matrix is composed of casein and glutaraldehyde which are hydrophilic biopolymer and crosslinker, respectively and their relative amounts in the CNPS are expected to affect extent of swelling and, consequently, the impregnation of iron oxide also.

### FTIR spectral analysis

**Figure 2:**
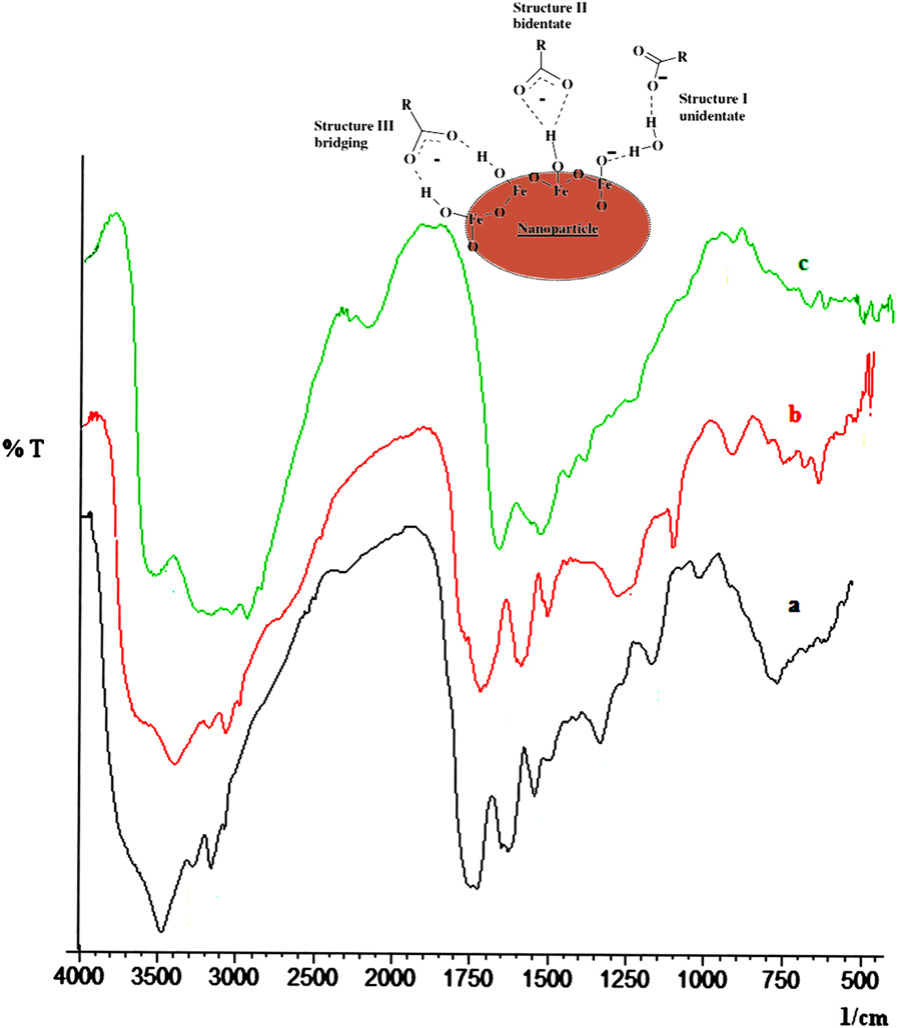
**FTIR spectra of a) native casein, b) CNPs, and c) IOICNPs.**

The FT-IR spectra of native casein, CNPs and IOICNPs are shown in (Figure 2a, b and c), respectively. Figure 2a shows absorption bands at 3455, 3100, 1661, 1530 and 1235 cm^−1^ which can be explained as follows: In the case of native casein, the amide A band at 3455 cm^−1^ and amide B at 3100 cm^−1^ are observed, which originate as a result of Fermi resonance between the first overtone of amide II and the N-H stretching vibration. Amide I and amide II bands are two major bands of the infrared spectrum of casein. The observed intense band for amide I appears at1661 cm^−1^ and is mainly associated with the C = O stretching vibration and depends on the backbone conformation and hydrogen bonding. The amide II bands obtained in the 1510 and 1580 cm^−1^ region result from the N-H bending and the C-N stretching vibrations. The obtained bands at 1661 cm^−1^ and 1531 cm^−1^ for the amide I and amide II, respectively also confirm the alpha helical structure of the casein protein.

Casein also exhibits another characteristic band at 1415 cm^−1^which may be attributed to the carboxylate group (O-C-O). As shown in **(**Figure 2b), a band appears at 1683 cm^−1^ and may be assigned to C = N stretching which confirms the presence of crosslinking between casein and glutaraldehyde. In (Figure 2c) the appearance of peaks around 450 and 480 cm^−1^ may be assigned to Fe–O bonds of magnetite, which are characteristic peaks of iron oxide (e.g., polyhedral Fe^3+^–O^2−^ )stretching vibrations of iron oxide, and thus confirm the impregnation of iron oxide into the matrix of casein nanoparticles [13,14].

According to Deacon and Phillips [15], the carboxylate ion may be coordinated to a metal atom in one of the following structures:

- **structure I:** unidendate complex where one metal ion binds with one carboxylic oxygen atom
- **structure II:** bidendate complex where one metal ion binds with two carboxylate oxygens
- **structure III:** bridging complex where two metal ions bind with two carboxylate oxygen’s. The FTIR spectra indicated the presence of two bands, 1415 cm^−1^ (V_s_: COO^−^) and 1538 cm^−1^ (V_as_: COO^−^), which may be attributed to the carboxylate ion of casein immobilized on the magnetite surface.

### SEM analysis

**Figure 3:**
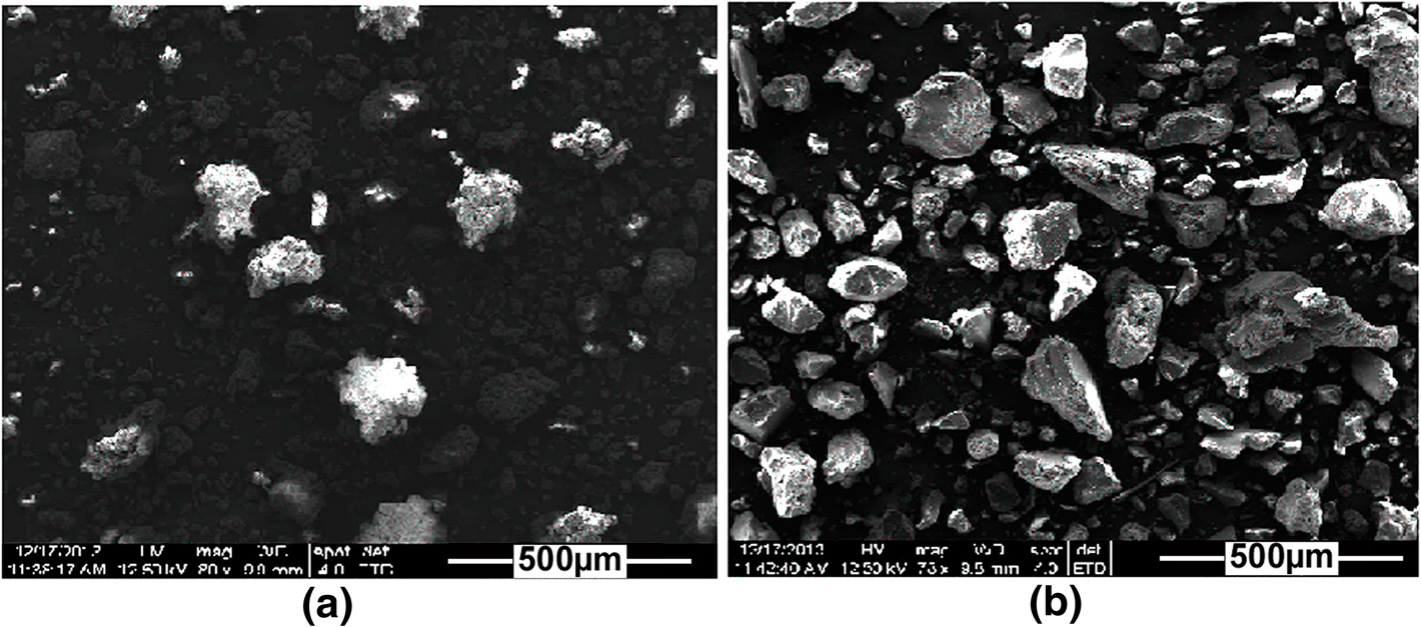
**SEM images of a) CNPs and b) IOICNPs.**

SEM images of CNPs and IOICNPs are shown in (Figure 3a and b), respectively which illustrate non-smooth morphology of CNPs and formation of iron oxide in the casein networks. The coating of iron oxide nanoparticles by the casein produces larger size particles due to the formation of the casein layers on the surfaces of iron oxide. During in-situ precipitation it may be inferred that iron oxides are assembled or attached inside the biopolymeric networks and on the casein surface as well. Loading of iron oxide inside the network affects its morphology and structural integrity. It is likely that the presence of intermolecular forces between casein macromolecular units facilitates formation of an extensive physical network of hydrogen bonds and other van der waal forces, which provide ‘nano’ domains for growth of the iron oxide nanoparticles as well as ensure their protection within the casein network. These biopolymeric networks may be considered as nanoreactors to construct or assemble iron oxide. The results may be attributable to contributions of a Fe-O^−^ coordination bond on the surface, steric effect and a compartment effect of the network structures of casein, which limit the growth of iron oxide, and thus play an important role in the process of the formation of iron oxide aggregates [16].

### TEM analysis

**Figure 4:**
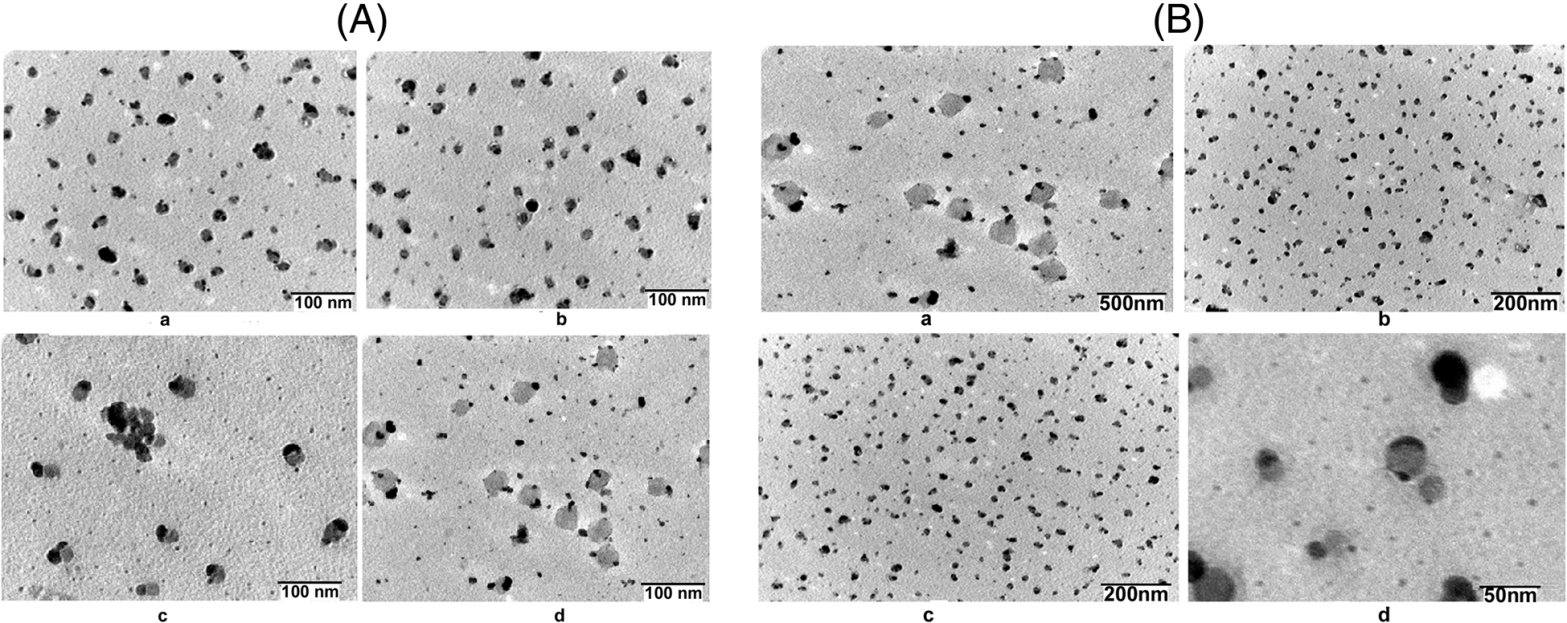
**TEM images of IOICNPs containing varying amount of casein 0.5 g, 1.0 g, 1.5 g, 2.0 g, and glutaraldehyde 5 mM, 10 mM, 15 mM and 20 mM. (A)** TEM images of IOICNPs containing varying amounts of casein a) 0.5 g, b) 1.0 g, c) 1.5 g, d) 2.0 g. **(B)** TEM images of IOICNPs containing varying amounts of glutaralehyde a) 5 mM, b) 10 mM, c) 15 mM, d) 20 mM.

In order to ascertain more precise morphology and size of the iron oxide particles at the nanoscale levels, TEM studies were performed. The size distribution of magnetic nanoparticles is an important parameter related to their biological applications and performance. Different shaped IOICNPs may be prepared by the facile co-precipitation method by adjusting the amounts of polymer, crosslinker and Fe^2+^/Fe^3+^ ratio, pH so as to investigate the influence on the shape and particle size of IOICNPs. It was observed that the synthesized IOICNPs displayed a relatively spherical distribution, good dispersion and a uniform morphology with distinct crystalline structure. From the TEM image of IOICNPs, it can be clearly demonstrated that magnetic nanoparticles comprise of core shell structure with homogenous incorporation of magnetite as a core of IOICNPs. The magnetic nanoparticles are homogeneously covered by the casein shells [17]. The particle size of IOICNPs may be controlled by the amounts of casein and glutaraldehyde. The TEM images of IOICNPs with different amounts of casein and glutaraldehyde are shown in Figure 4A and B, respectively, which indicate that an average size of IOICNPs falls in the range of 80–90 nm. As the amount of casein increases from 0.5 to 2.5 g, the size of the as-prepared IOICNPs increases from 15 nm to 50 nm as shown in (Figure 4A). The results also show that increasing amount of casein tends to produces largerer particle size because it can produce a bigger micelle [10,17]. Thus, on increasing the amount of casein, the size of IOICNPs also increases. The average particle size of the nanoparticles, as a function of the oil/water ratio in the emulsions, decreases from 20 to 8 nm with increasing oil/water ratio from 1.3 to 7.0. When the oil/water ratio is high, the molar ratio of casein to water is also increased, resulting in a high surface tension at the oil/water interface. This produces small water droplets and determines the size of iron nanoparticle [18]. The effect of glutaraldehyde concentration on the size of IOICNPs is shown in (Figure 4B), which reveal that as the concentration of crosslinker increases, the number of more crosslink points increases thus resulting in an increase the crosslinking density. As a result, the network voids are minimized and the particle size decreases.

The Figures also show that the nanoparticles formed are present as aggregates due to the reason that they have a natural tendency to undergo clustering due to variety of charges and functional groups present on their surfaces.

### Raman spectral analysis

**Figure 5:**
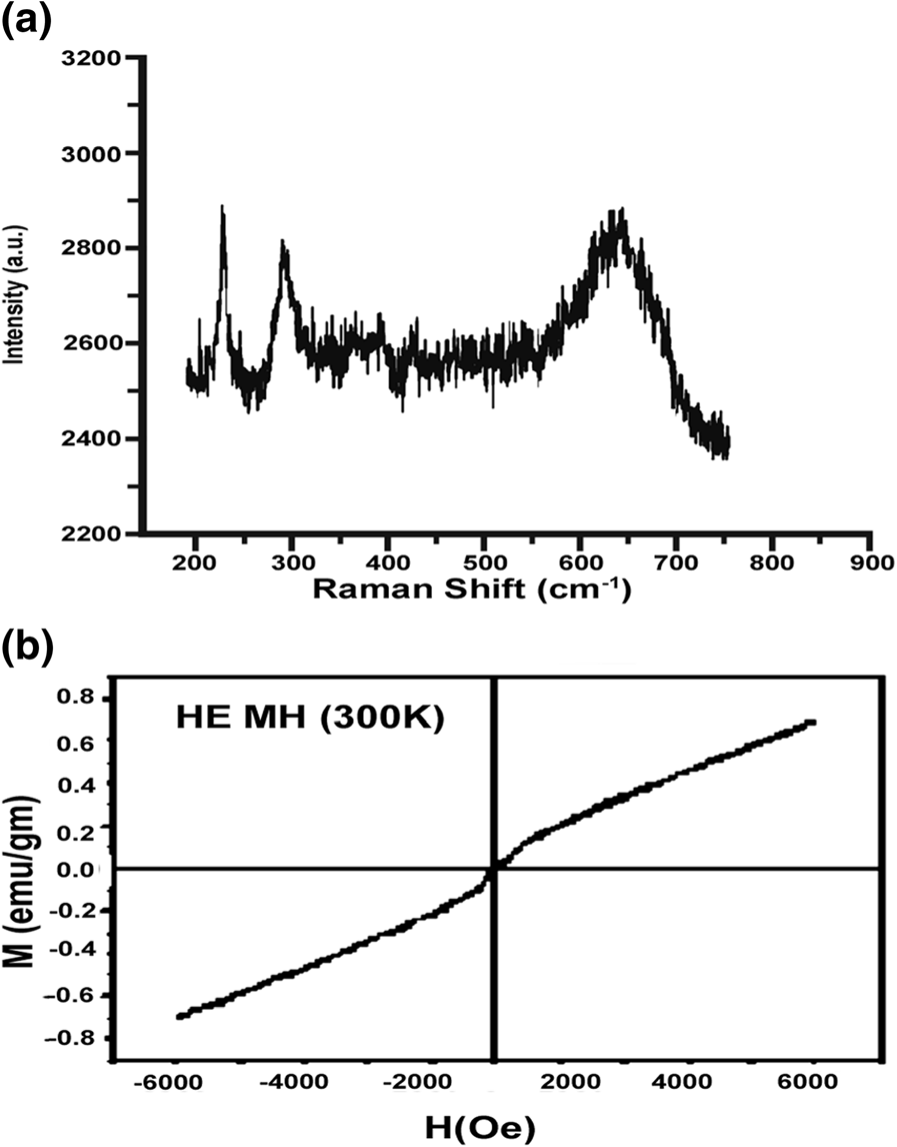
**Raman spectra and VSM (M-H) curve of IOICNPs at 300K. a)** Raman spectra and **b)** VSM (M-H) curve of IOICNPs at 300 K.

Raman spectra of IOICNPs are shown in (Figure 5a), which show characteristic Raman bands for casein due to amide I (CONH) at ~1666 cm ^−1^ and amide III band at ~1245 cm ^−1^. Between these Raman bands an intense peak is observed at 1450 cm ^−1^, which is attributed to the CH_2_ scissoring mode. In the Raman spectra weaker peaks observed at 193 cm^−1^, 306 cm^−1^ and 538 cm^−1^ confirm the presence of iron oxide in the form of magnetite. Moreover, an additional strong peak is also observed at 668 cm^−1^. For correct assignment of sample combined Raman data key can be used as follows [19,20].

(i) **Fe**_**3**_**O**_**4**_**:** 193 (weak), 306 (weak), 538 (weak), 668 (strong),
(ii) **gFe**_**2**_**O**_**3**_**:** 350 (strong), 500 (strong), 700 (strong); and
(iii) **aFe**_**2**_**O**_**3**_**:** 225 (strong), 247 (weak), 299 (strong), 412 (strong), 497 (weak), 613 (medium).

### VSM analysis

The magnetization versus magnetic field plot (M-H magnetization curve and hysteresis loop) at 300 K, for the impregnated casein nanoparticles was measured over the range of applied field between −6000 to +6000 Oe with a sensitivity of 0.1 emu/g, using vibrating sample magnetometer. The results are shown in (Figure 5b) which show that the saturation magnetization value is around 64 emu g^−1^, and the hysteresis is very weak. The value obtained is lower than the reported value of 92–100 emu g^−1^ for magnetite nanoparticles and may be attributed to the fact that below a critical size, nanocrystalline magnetic particles may be of single domain and show unique phenomenon of superparamagnetism [21,22]. The reduction in saturation magnetization of Fe_3_O_4_ particles may be attributed to the presence of non-magnetic layer on the surface of the particles, charge distribution, super-paramagnetic relaxation and spin effect because of ultrafine nature of the particles.

### XPS analysis

**Figure 6:**
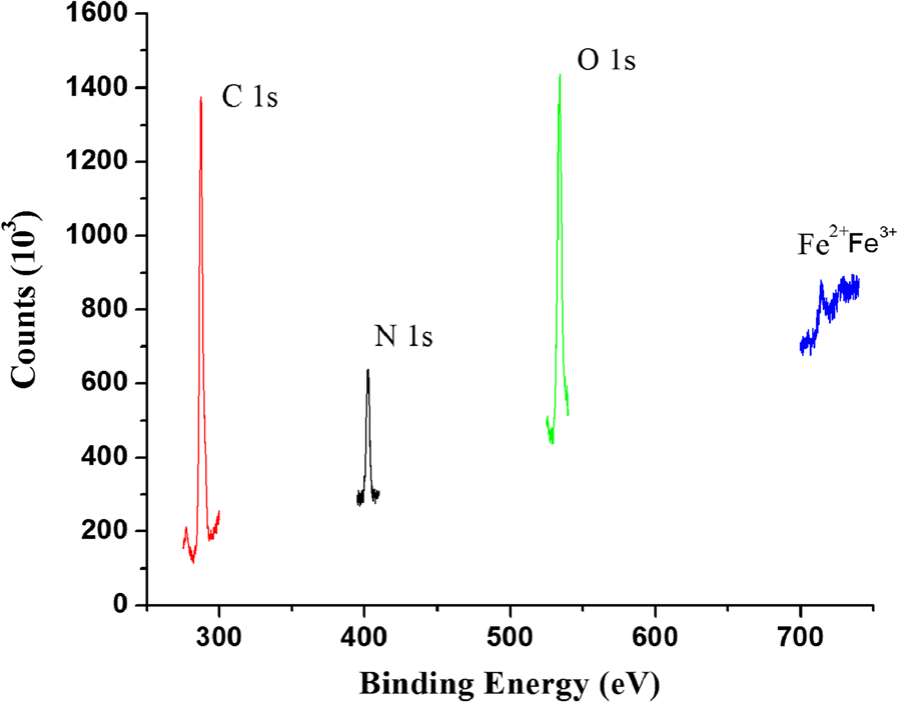
**XPS of IOICNPs.**

In the present study XPS analyses were done to monitor the iron oxide deposition in the cross-linked IOICNPs. The XPS spectra of the cross-liked IOICNPs is shown in the (Figure 6) Which reveal the presence of the C 1 s, O 1 s, and N 1 s core-level peak. However, after impregnation of iron oxide, the spectrum exhibits two more peaks associated with Fe^2+^ and Fe^3+^, due to the iron oxide deposition. It is worth to mention that the peak assignment is based on characteristic binding energies reported in the literature [23,24]. Furthermore, the O 1 s core-level spectra of the cross-linked IOICNPs were fitted using two peaks at 532.3 eV and 534 eV. The first one is associated with the binding energy of the [C = O] in the imide group and carboxylic acid group while the second one at the binding energy of the OH in the carboxylic acid group. The absence of the peak at 287.2 eV, associated with the binding energy of carboxylic acid groups, is accompanied by an increase in the intensity of the peak at 285.9 eV, due to the contribution of the carboxylate species in the crosslinked biopolymer [25]. These groups were also observed in the FTIR analysis. The O 1 s core-level spectrum from the resulting composite was fitted to peaks at 530.2 eV (g-Fe_2_O_3_), 531.4 eV (a-FeOOH). Furthermore, the spectrum displays two peaks associated with the iron oxide in the composite which are in good agreement with the magnetite, at 715.3 eV and 725.4 eV for Fe^2+^ and Fe^3+^ ions.

The XPS was applied to provide elemental information of surface composition of IOICNPs after Fe_3_O_4_ loading. There were C, N, O and Fe elements in the magnetic IOICNPs, which further proved that Fe_3_O_4_ nanoparticles were in situ synthesized in the IOICNPs [26]. The different oxidation states of the iron in these nanoparticles can also be detected and distinguished from each other by XPS.

### In vitro cytotoxicity test

**Figure 7:**
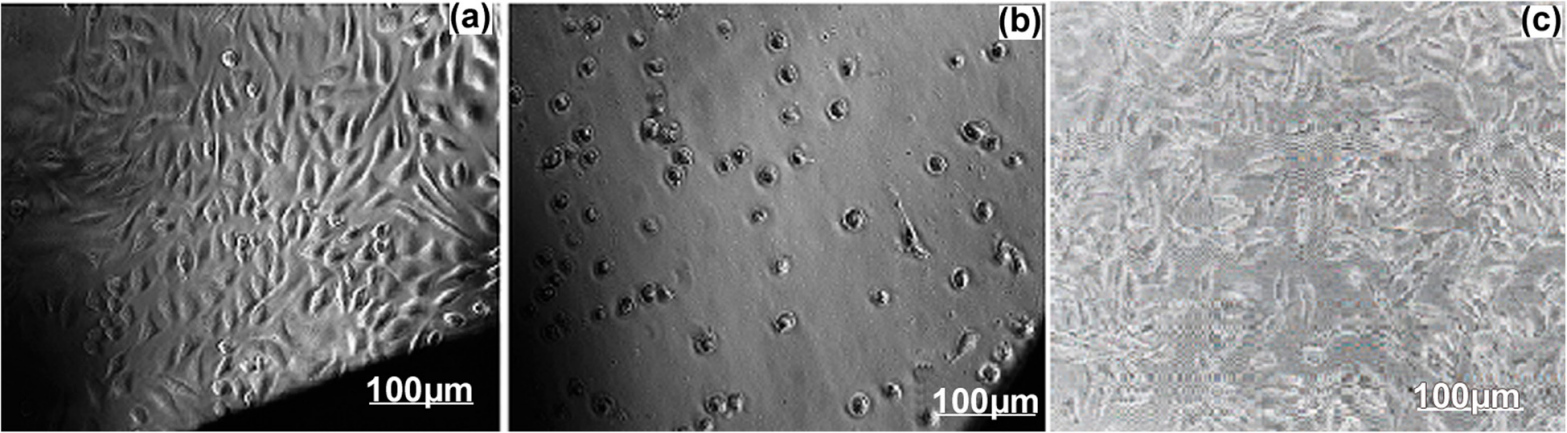
**MIcroscopic images showing L-929 cells. a)** negative control, **b)** positive control, and **c)** IOICNPs, respectively.

In order to determine in vitro cytotoxicity of the prepared materials Test on extract method was performed (ISO10993-5, 2009). In this method powdered (0.2 g) material was soaked in culture medium (1 mL) with serum and then the extract was prepared by incubating the presoaked test material with serum for 24 h. After incubation, the extract was filtered using 0.22 μm millex gp filter. 100% extract were diluted with culture medium to get 50% and 25% concentrations. Different dilutions of test sample extracts, positive control and 100% extracts of negative control in triplicate were placed on subconfluent monolayer of L-929 cells. After incubation of cells with extracts of the test sample and controls at 37 ± 1°C for 24 to 26 h, culture was examined microscopically for cellular response. For negative control the sample was prepared by incubating 1.25 cm^2^ polyethylene disc with 1 mL culture medium with serum at 37 ± 1°C and positive control was prepared by diluting phenol stock solution (13 mg/mL) with culture medium with serum. The cytotoxicity reactivity were graded based on zone of lysis, vacuolization, detachment and membrane disintegration as 0, 1, 2, 3 and 4 representing none, slight mild, moderate and severe, respectively. The quantitative evaluation of reactivity for negative and positive controls and test sample are summarized in Table 1, while microscopic observation are depicted in (Figure 7a, b and c), respectively.

**Table 1:** **Quantitative evaluation of in vitro cytotoxic reactivity of various samples**

**S. no.**	**Sample**	**Grade**	**Reactivity**
**1.**	**Negative control**	**0**	**None**
**2.**	**Positive control**	**4**	**Severe**
**3.**	**IOICNPs**	**0**	**None**

It was found that the test sample showed none reactivity to fibroblast cells after 24 h of contact. The achievement of numerical grade more than 2 is considered as cytotoxic effect. Since the polymer network material in the present work achieved a reactivity grade less than 2, the material is considered as not cytotoxic.

### Effect of Magnetic Field (MF) on the swelling

**Figure 8:**
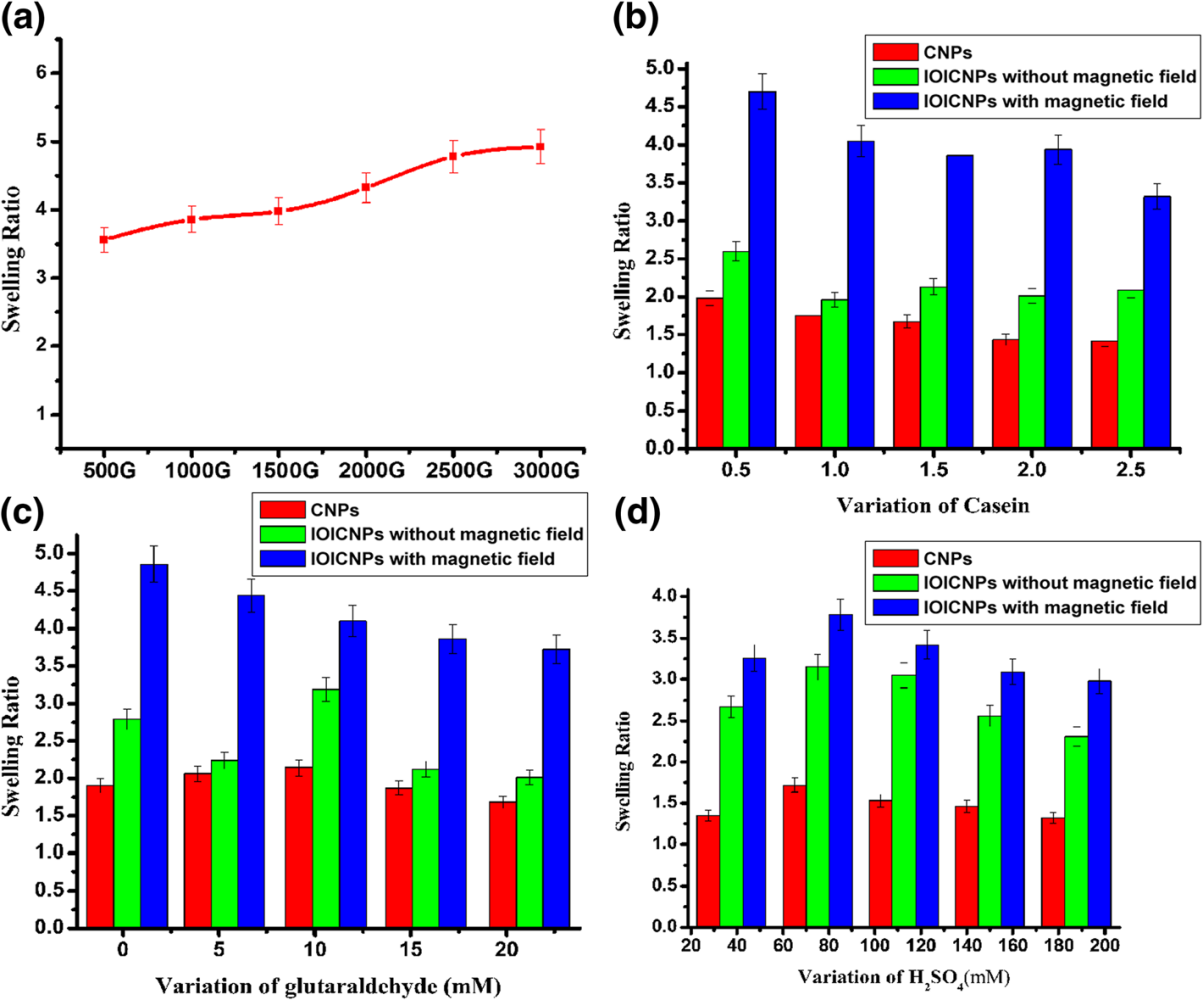
**Effect of magnetic field, casein, glutaraldehyde, and H**_**2**_ **SO**_**4**_ **on swelling of CNPs and IOICNPs. a) magnetic field,** **b)** casein, **c)** glutaraldehyde, and **d)** H2SO4 on swelling of CNPs and IOICNPs.

In magnetic drug targeting swelling-controlled system must have satisfactory swelling properties and high capacity of drug loading. The degree and time of swelling are important characteristics and have significant effect on the release kinetic of loaded drugs from swelling-controlled systems. To evaluate the effect of MF the on the swelling of IOICNPs, the MF was varied in the range of 1000 to 3000 G. The results are depicted in (Figure 8a) which reveal that the swelling ratio increases with increasing strength of magnetic field. The observed results may be attributed to the fact that the magnetic moment of a material **M** is proportional to the applied field H.

7 $$ \mathbf{M}=\boldsymbol{\upchi} \mathbf{m}\mathbf{H}, $$

where χm is magnetic susceptibility of the material. The possible reason for the observed increased swelling upon application of external magnetic field may be that under the applied field the magnetic nanoparticles get aligned with the applied field. Since the particles are in constant motion, they will experience fluctuating magnetic field which may cause agitation of the nanoparticles. This will produce a motion in the nanoparticles matrix and result in relaxation of polymer chains of the nanocomposite, thus, leading to a greater swelling of IOICNPs. This may also enlarge the nanostructure of the polymeric matrix to produce porous channels that cause enhanced diffusion process enabling easy swelling. The mechanical deformation generates compressive and tensile stresses, enhancing the penetration of water molecules. Similar type of result has also been reported elsewhere [27].

### Effect of chemical composition in CNPs and IOICNPs

In the present work, the influence of chemical composition of casein nanoparticles on their swelling ratio has been investigated by varying the amounts of casein and crosslinker (glutaraldehyde), in the feed mixture, respectively. The observed results may be discussed as below:

### Variation of casein in CNPs and IOICNPs

The effect of increasing biopolymer content on the swelling characteristics of CNPs and IOICNPs has been investigated by varying the amount of casein in the range 0 · 5–2.5 g while keeping the concentration of glutaraldehyde as constant. The results are shown in (Figure 8b), which clearly indicate that the swelling of IOICNPs is higher than CNPs and initially increases up to 0.5 g of casein content and thereafter decreases with further increase in the amount of casein. The results may be attributed to the fact that till 1.5 g of casein, highly compact nanoparticles are formed which restricts the inward movement of water molecules thus resulting in a decrease in swelling ratio. However, beyond 1.5 g of casein, swelling ratio is found to increase with increase in the amount of casein up to 2.5 g. The observed results are due to the fact that casein is a hydrophilic biopolymer and its increasing amount in the particles will obviously enhance the hydrophilicity of the nanoparticles and, thus, an increase in the swelling ratio is obtained.

In IOICNPs, the decrease in the total water content , might be attributed to effective interactions between the iron oxide and the polymer matrix. These interactions may arise from the carboxylic groups of casein that act as iron-binding sites [28,29]. Taking into account that the number of carboxylic groups increases on increasing the amount of casein, and the interactions between iron oxide and polymer matrix a decrease in the swelling behavior of IOICNPs may be justified.

The results also indicate that swelling of nanoparticles in magnetic field is higher than that in the absence of magnetic field. The results can be explained by the fact that due to the applied magnetic field, the magnetic moments of impregnated iron oxide nanoparticles tend to get aligned with the external magnetic field and while doing so, they produce a motion of macromolecular chains in the casein matrix. Thus due to the mobility of iron oxide nanoparticles, the macromolecule chains of casein get relaxed and facilitate greater inclusion of water molecules into the biopolymer matrix [30]. This clearly results in an enhanced swelling of nanoparticles.

### Variation of glutaraldehyde in CNPs and IOICNPs

The effect of crosslinker on the swelling profiles of CNPs and IOICNPs have been investigated by varying the concentration of glutaraldehyde in the range 0.02 to 20 mM. The results are shown in (Figure 8c), which clearly reveal that the water sorption by nanoparticles constantly increases while beyond 10 mM concentration a drop in swelling ratio is observed.

The observed increase may be attributed to the fact that glutaraldehyde is a low molecular weight crosslinking agent, and it crosslinks by reacting with the NH_2_ groups of casein at its two terminals. Thus, a crosslinked network is developed as a result of crosslinking reaction between casein and glutaraldehyde thus creating a wide space in its structure and, therefore, possessing high capacity of accommodating water molecules into the network [31]. In this way, the capacity of nanoparticles to accommodate large number of water molecules results in an increased swelling ratio. The decrease observed beyond 10 mM of glutaraldehyde may be explained by the reason that much higher crosslinker content in the nanoparticles matrix reduces the free space in the nanoparticles network accessible to the penetrating water molecules and consequently results in a fall in the swelling capacity.

Some authors have also reported that introduction of crosslinker in to the polymer matrix enhances its glass transition (T_g_), which because of glassy behavior of polymers restrains the mobility of network chains and, thus decreases the swelling [32]. The swelling results also indicate that the applied magnetic field also enhances the swelling of nanoparticles which has already been explained earlier.

### Variation of Sulphuric acid

Ionically crosslinked CNPs were successfully prepared by a two-step process. The first step involved the formation of oil droplets (emulsion) by an oil-in-water emulsion formation. The second step was the solidification by using sulphuric acid of the formed droplets by ionic crosslinking of casein (pH 2) enveloping the oil droplets with glutaraldehyde . By decreasing the pH of casein solution below its isoelectric point (4.6–4.8), the amino groups of casein become positively charged by protonation and can strongly attract the negatively charged aldehydic groups of glutaraldehyde, leading to the formation of ionically crosslinked nanoparticles [33].

The effect of solidifying agent (sulphuric acid) on the swelling profile of CNPs and IOICNPs has been investigated by varying the concentration of H_2_SO_4_ in the range 20–200 mM. The results are shown in (Figure 8d), which clearly reveal that the swelling of nanoparticles constantly increases while beyond 80 mM concentration a drop in swelling behavior is observed. The observed decrease could be attributed to the fact that as the amount of H_2_SO_4_ increases in the feed mixture, the number of positively charged amino groups increases which enhances the interaction between aldehydic group ( ^**−**^**O-**^**+**^**C-H** ) increases thus resulting high crosslinking density between casein and glutaraldehyde.

### Variation of Fe^2+^/ Fe^3+^

**Figure 9:**
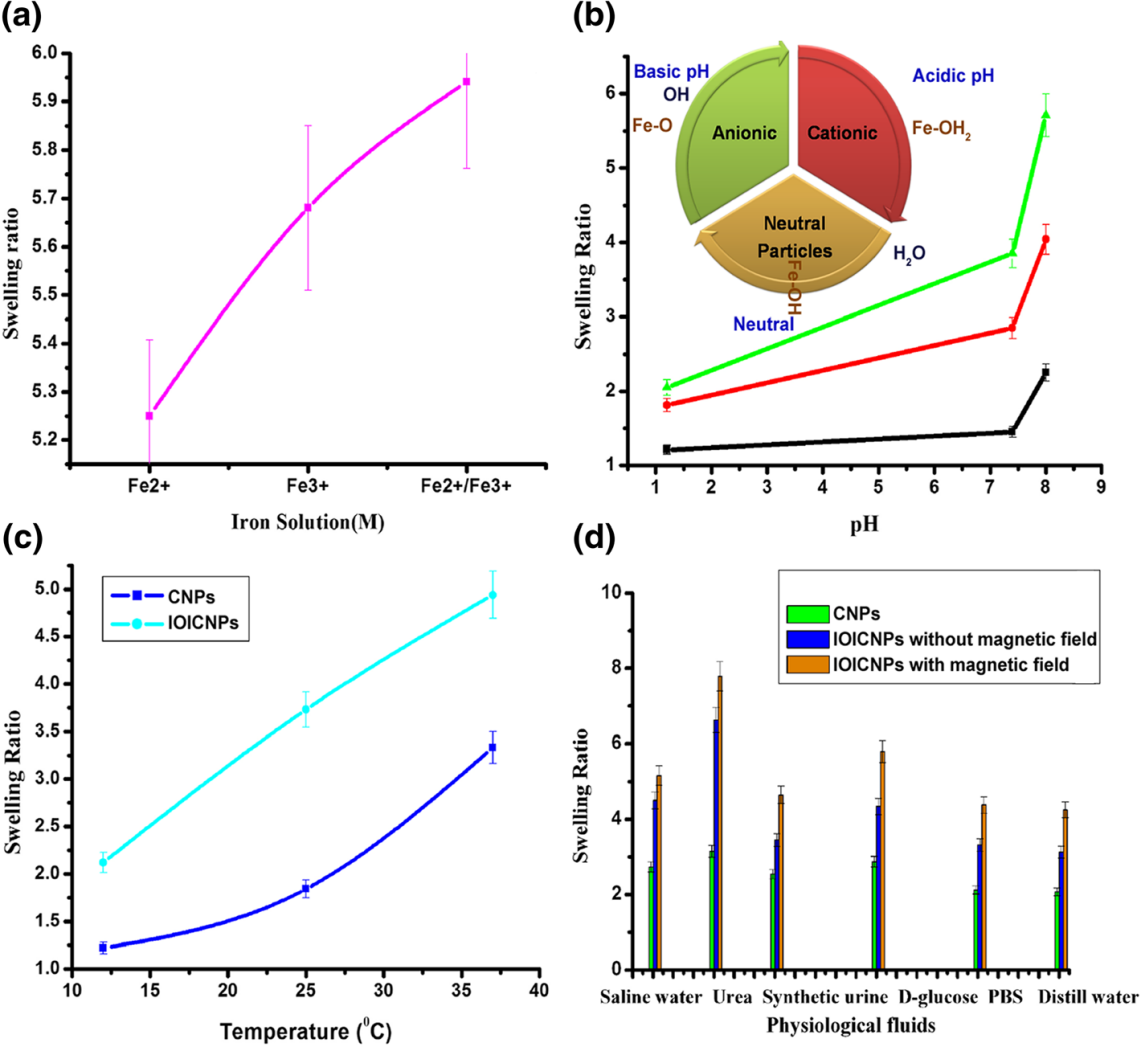
**Effect of iron salts, pH, temperature and simulated biofluids on swelling of CNPs and IOICNPs. a)** iron salts, **b)** pH, **c)** temperature, and **d)** simulated biofluids on swelling of CNPs and IOICNPs.

The CNPs treated with Fe^2+^/Fe^3+^ solution contain iron ions dispersed throughout the casein network. The electrostatic forces between the iron ions and amide groups of casein are responsible for the increase in the swelling of IOICNPs, as compared to CNPs without ions. When the iron ions **(Fe**^**2+**^**0.5/Fe**^**3+**^**1 M)** loaded particles are treated with alkali, magnetite nanoparticles are formed inside the CNPs. This results in an increased swelling capacity due to increased electrostatic forces between the amide groups and iron oxide. The results are shown in (Figure 9a).

The swelling capacity follows the order CNPs < IOICNPs which has same trend as observed in PAM hydrogel–silver nanocomposites [34]. When the iron ions loaded nanoparticles are treated with NaOH, magnetite nanoparticles are produced inside the matrix. The particles treated with iron salts had iron ions dispersed throughout the polymeric network. The electrostatic forces between the iron ions and amide groups of casein matrix are responsible for the increase in the swelling of iron ion loaded nanoparticles, as compared to native casein nanoparticles without ions.

Fe_3_O_4_ magnetic nanoparticles were prepared with different concentrations of Fe^2+^, Fe^3+^ in aqueous phase, while other preparation conditions were kept same. The average size of Fe_3_O_4_ magnetic nanoparticles increases as concentration of iron salt solution increases. The particle size was found to increase with the increase in concentration of iron salts [35].

### Effect of pH

pH sensitive macromolecular devices have been most frequently used to design swelling controlled release drug formulations for oral administration which is the most clinically acceptable way of drug delivery. In the present study, the effect of pH on the swelling ratio of CNPs and IOICNPs has been studied by adjusting pH of swelling medium in the range of 1 · 0 to 9.0. The results are depicted in (Figure 9b), which clearly indicate that the swelling ratio of particles constantly increases with increase pH. The increase observed in the swelling ratio of the particles with increasing pH may be explained as follows. It is known that casein is cationic in nature and therefore a change in pH of the swelling medium also affects the charge profiles of casein as well as IOICNPs. The swelling results indicate a significant dependence of swelling behavior of the nanoparticles on different pH values. In both the cases of CNPs and IOICNPs, the results reveal that at pH 1.8, a lower swelling ratio was observed, because PK_a_ of casein is about 4.2 and most of the carboxyl groups in the casein exist in the form of COOH at low pH medium (pH 1.7). In the macromolecular nanoparticle network of the hydrogen bonding produced by –COOH groups of casein led to the stronger interaction between polymer chains. Accordingly, the swelling ratio in pH 1.7 is relatively lower. At higher pH, the carboxylic groups get ionized and acquire –COO^−^ form. Thus, weak hydrogen bonding between biopolymer chains and electrostatic repulsion between –COO^−^ groups result in the higher swelling ratio [36].

Since pKa of casein is 4.2, the species involved in the interactions are NH_3_^+^ and COOH at pH 1–3, NH_2_ and COO − at pH 7–13. In acidic conditions, the swelling is controlled mainly by the amino group (NH_2_) which is a weak base with an intrinsic pKa value of about 6.2. So, it gets protonated and the increased charge density on the biopolymer enhances the osmotic pressure inside the network particles because of the NH_3_^+^-NH_3_^+^ electrostatic repulsion. This osmotic pressure difference between the internal and external solutions of the network is balanced by the swelling of the network. However, under very highly acidic conditions (pH < 3), a screening effect of the counter ions, i.e. Cl^−^, shield the charges of the ammonium cations and prevent an efficient repulsion. As a result, a remarkable decrease in equilibrium swelling is observed. Similarly, the screening effect of the counter ions (Na^+^) limits the swelling at pH > 8.5 [37].

The swelling of IOICNPs is governed by negative charge possessed by iron oxide nanoparticles. When pH of the swelling medium is 9.0, the number of negatively charged groups (**Fe-O**^**−**^**)** is large, so the swelling is maximum because of the strong electrostatic repulsion between negatively charged groups. When pH of the swelling medium is 7.0, the protons from the swelling medium neutralize most of the negatively-charged groups and, therefore, the swelling ratio decreases due to the reduced electrostatic repulsion. At 1.2 pH, all negatively-charged groups are neutralized; instead, there would be some positively charged amine groups and iron species **(Fe-OH**_**2**_^**+**^), because the amine groups in the IOICNPs are fewer than the carboxyl groups and the net charge is quite low, thus that the swelling is also very low [38].

When pH is less than isoelectric point (pI) of casein, the extent of increase in swelling ratio at acidic medium is small, because there are very few amine groups existing along protein chains so that the positive charges are very limited. The swelling becomes minimum when pH of the medium is close to the pI of the casein (4.6-4.8). This is because the net charge on casein molecules is close to zero at pI, which means almost no electrostatic repulsion exist between the casein chains. On the other hand, when pH > pI, there are lots of negatively-charged groups on the casein molecule, which results in an increased swelling ratio. The higher the pH, greater is the surface charge and consequently the higher electrostatic repulsive forces result in higher swelling ratio [39].

### Effect of temperature

In the present study, the effect of temperature on swelling was studied by varying the temperature in the range 12.5–37.5°C. The results are shown in (Figure 9c), which indicate that with increase in temperature, the swelling of nanoparticles increases from 2.5 to 4.5 in the whole studied range of temperature. The observed increase in the swelling of IOICNPs may be explained by the fact that a rise in temperature enhances the rate of diffusion of water molecules and segmental mobility of biopolymer chains which results in a greater degree of swelling [40]. However, beyond 30°C the swelling ratio decreases, which may be due to the reason that at higher temperature the hydrogen bonds holding water molecules and the polymer chains get broken and, therefore, the swelling ratio decreases.

### Effect of physiological fluids

The effect of nature of physiological fluids on the swelling of CNPs and IOICNPs in absence and presence of magnetic field has been investigated by performing swelling experiments in various simulated physiological fluids like urea, D- glucose, PBS, saline water, distilled water and synthetic urine. The results are presented in (Figure 9d), which indicate that swelling ratio is quite high in urea in comparison to other fluids and lower degree of water sorption is noticed. The possible reason for the higher swelling ratio in urea may be that the presence of urea works as hydrogen bond breaker in casein macromolecule and this tends to result in greater relaxation of biopolymer chains which eventually leads to higher swelling. In the case of other fluids the salt ions in the medium lowers the osmotic pressure difference between the casein matrix and solvent medium which causes a decrease in swelling ration of nanoparticles.

The change in swelling due to the presence of electrolyte concentration has also been predicted theoretically. Fernández-Nieves et al. [41] demonstrated that the addition of salts modifies the ionic distribution inside the casein matrix, altering the number of dissociated groups in the network, and changing the net charge. The change in the network charge state makes particles swell or shrink, depending on whether the electrical repulsions increase or decrease, respectively.

## Conclusions

The controlled size magnetite nanoparticles have been successfully prepared via a convenient co-precipitation method and characterized by various analytical techniques, such as FTIR spectroscopy, TEM, SEM, XPS, VSM and Raman analyses which confirm the *in situ* impregnation of nanosized iron oxide within the matrix of casein nanoparticles. The biopolymeric nanoparticles clearly show the presence of characteristic functional groups of casein and iron oxide as confirmed by their FTIR spectra. SEM and TEM of the nanoparticles provide information about their size and morphology.

The IOICNPs show an optimum swelling when the casein content is 0.5 g while on increasing casein content further the degree of swelling decreases. Likewise, when the concentration of crosslinker increases from 0.02-20 mM, the quantity of water imbibed by the nanoparticles increases while beyond 10 mM of crosslinker concentration, the extent of swelling decreases. It is also found that in alkaline pH the nanoparticles show an enhanced swelling which thereafter decreases with further increase in pH. In the case of rising temperature the swelling ratio constantly increases. The prepared IOICNPs exhibit property of superparamagnetism which is a significant for biomedical applications. It is also observed that the swelling of nanoparticles is enhanced by the application of external magnetic field. Thus the present swelling system may be helpful in designing targeted drug delivery carriers using external magnetic field.
